# Muslim nurse’s spiritual sensitivity as a higher perception and reflection toward spiritual care: a qualitative study in southeast Iran

**DOI:** 10.1186/s12912-022-01044-4

**Published:** 2022-10-05

**Authors:** Omolbanin Akbari, Mahlagha Dehghan, Batool Tirgari

**Affiliations:** 1 Nursing Research Center, Kerman University of Medical Sciences, Kerman, Iran; 2 Department of Critical Care Nursing, Razi Faculty of Nursing and Midwifery, Kerman University of Medical Sciences, Kerman, Iran; 3 Department of Medical Surgical Nursing, Razi Faculty of Nursing and Midwifery, Kerman University of Medical Sciences, Kerman, Iran

**Keywords:** Spiritual sensitivity, Nurses, Muslim, Qualitative research, Iran

## Abstract

**Background:**

Spiritually sensitive nurses perceive the spiritual attitudes and feelings of others. They play a positive role in providing spiritual care to patients. Spiritually sensitive nurses deal appropriately with suffering, frustration, and spiritual dysfunction. Therefore, the present study aimed to explain Iranian nurses’ experiences of spiritual sensitivity.

**Methods:**

This qualitative descriptive explorative study used conventional content analysis and purposeful sampling to explain the experiences of Iranian nurses (n = 19). This study used in-depth semi-structured interviews with 19 nurses, as well as maximum variation sampling to gather rich information (age, sex, religion, work experience, level of education, marital status, type of hospital and ward) from March 2021 to January 2022. The current study also employed Guba & Lincoln criteria to increase data trustworthiness and Graneheim and Lundman approach to analyze the content.

**Results:**

The research data showed 497 codes, 1 theme, 3 categories, and 6 subcategories. The theme of “Nurse’s spiritual sensitivity as a higher perception and reflection toward spiritual care” included three categories of the spiritual and professional character of the nurse, perception of the spiritual needs of patients and their families, and the nurse’s reflection on the religious beliefs of patients and their families.

**Conclusion:**

Spiritual sensitivity helps a nurse to provide holistic care for patients and their families. Therefore, managers and policymakers should create guidelines to help nurses become more spiritually sensitive as well as to meet spiritual needs of patients. Further quantitative and qualitative research should confirm these results in other social and cultural contexts.

## Background

Holistic nursing refers to nurses’ knowledge, skills, and theories to deliver care and engage in therapeutic communication [1]. Holistic care meets physical, psychological, social, and spiritual needs in patients [2]. Holistic nursing takes into account the beliefs and values of each person [1]. Spirituality seeks meaning and purpose in human beings as well as perceives life and natural forces in the world. Thus, concepts, such as spirit and spirituality can have a variety of definitions [3]. Every person has their own definition of spirituality influenced by their personal beliefs and value systems [4]. Spirituality, according to Joseph et al. (2017), is a broad, unstructured, personal, and natural phenomenon in which one seeks a connection with a greater force or objective [5]. Spirituality has been identified as the essence of being human [6]. Healthcare professionals believe that spirituality is a vital aspect of healthcare [7]. Spiritual nursing aims to address the patient’s anxieties, concerns, and suffering to relieve anxiety, promote hope, and enable patients to attain inner peace [8]. Nurses perform spiritual interventions based on ethical principles; therefore, spiritual care can be considered as a moral issue and nurses must be spiritually sensitive to provide spiritual care [9].

The term “spiritual sensitivity” refers to the ability to see and understand the spiritual perspectives and experiences of others [9]. The structure of spiritual sensitivity is one in which the visible actions of people are in harmony with their spiritual ideals. According to Straś-Romaniowska et al. (2016), sensitivity covers values, ideals, emotions, and the results of activities performed [10]. Spiritual sensitivity involves considering spiritual values in a paradoxical context and being conscious of one’s tasks and obligations within that context. Spiritual sensitivity identifies right and wrong easily and leads to performing the correct thing [11]. Kazemi et al. (2021) defined spiritual sensitivity as the attention to the spiritual values of a situation and responsibility for that situation[12]. Callahan (2018) believed that spiritual sensitivity was necessary to convey spiritual competence [13]. Spiritual sensitivity allows us to be aware of religious and spiritual issues and to respond to individual’s faith-based concerns [14]. Spiritual sensitivity is a key aspect of holistic care. Spiritual sensitivity makes it possible to respond to the needs of patients better. Spiritual sensitivity improves relationships between health care providers and patients and increases patient satisfaction [12]. Therefore, In addition to spiritual knowledge, healthcare professionals require spiritual sensitivity to face spiritual and moral obstacles in their everyday work [15].

The review of literature showed that the definitions provided were general definitions of spiritual sensitivity and were not specific to nurses [10, 16, 17]. These definitions focus on the values and sense of responsibility for them. On the other hand, most studies emphasized the importance of spiritual sensitivity and mentioned general definitions. The following questions might help to clarify the key problems concerning spiritual sensitivity: what exactly does spiritual sensitivity in nursing mean? What are its features? What are the examples of spiritual sensitivity? What circumstances cause spiritual sensitivity to manifest?. It is clear that concepts find meaning within social contexts, and the meaning of a concept varies from context to context; therefore, a detailed exploration of a concept requires local, cultural, social and managerial factors [18]. It is vital to explore spiritual sensitivity of nurses in the healthcare system based on the lived experiences of field participants. Iran is a religious country with 99% of its people being Muslim [19]. People with different religions live in Iran. Most Iranians are Shiite (80–90%), and the next popular religion is Sunni (5–9%). Sistan and Baluchestan, a province at southeastern Iran, has a high Sunni population [20]. The present study aimed to explore nurses’ experiences of spiritual sensitivity in southeastern Iran.

## Methods

### Study design and setting

This qualitative descriptive-explorative study used conventional content analysis, a qualitative method for analyzing written, oral, or visual communication messages. The purpose of content analysis study is to obtain a brief and comprehensive description of the phenomena [21]. The current study tried to analyze the latent content as well as the manifest content. This study was conducted in Zahedan, Sistan and Baluchistan province, southeastern Iran. The inhabitants are both Sistani and Baluch, as well as Muslim, but their dialects and religions differ. The Sistani people speak Persian and are Shiite, while Baluchi people are Sunni [22]. Although they are different in religion and language, they coexist and interact with each other. The healthcare system also employs nurses of both ethnicities to provide care to patients.

### Sampling, participant, and data collection

The present study interviewed nurses working at hospitals affiliated with Zahedan University of Medical Sciences (ZUMS), as well as nurses from Corona centers and semi-public hospitals affiliated with other organizations. Inclusion criteria were nurses with a bachelor’s degree or a higher degree in nursing, clinical work experience, and a desire to express their experiences. The current study used purposeful sampling to select participants and interviewed nurses to obtain rich and varied information. The current study selected various personal and occupational characteristics, such as sex, age, religion, marital status, level of education, work experience, position, type of hospital (public, semi-public), and type of wards to provide a wide range of information. Fifteen female and four male nurses were available. The nurses’ ages ranged from 25 to 55 years, and their work experience ranged from 1 to 30 years (Table 1). The method of data collection was interviews and field notes. The researcher first visited participants and explained the research objectives. She interviewed participants in their workplace. All participants participated in the study and wanted to share their experiences. The first researcher conducted open-ended, semi-structured, in-depth and individual interviews to collect data. Open-ended questions in the interview guide allow respondents to explain their own experiences and follow-up questions were the result of the participants’ responses. Table 2 showed questions that reflected participants’ experiences of spiritual sensitivity. The researcher audio-recorded and transcribed 30-75-minute interviews verbatim. The researcher continued sampling until data saturation meaning that new information was unavailable. The researcher reached data saturation after 22 interviews with 19 participants. She also used and recorded field notes in different shifts in the hospital. In general, she conducted 22 interviews and 6 field notes with 19 participants. She re-interviewed three participants to clarify some of their statements. She did sampling from March 2021 to January 2022.

**Table 1:** **Participants’ characteristics (N = 19)**

Participants		
**Sex**	Male	4
	Female	15
**Age (yr)**	Minimum	25
	Maximum	55
**Religion**	Shiite	10
	Sunni	9
**Marital status**	Single	5
	Married	14
**Work experience**	Minimum	1
	Maximum	30
**Level of education**	Bachelor’s degree	10
	Master’s degree	6
	PhD	3
**Religiosity**	None	4
	Low	4
	Moderate	5
	High	6
**Spirituality**	None	-
	Low	2
	Moderate	9
	High	8
Type of ward*	Emergency	13
	Medical	7
	ICU	10
	Operating room	1
	CCU	6
	Coronary	3
	Angiography	2
	Hematology	2
	Infection	4
	Surgery	7
	Nursing management office	3
	Orthopedics	1
	Psychiatrics	2
	Gynecology	2
	Pediatrics	6
	Dialysis and nephrology	4

^*^Some participants had work experience in different hospitals and wards

**Table 2:** Examples of questions

1. As you have provided care for different patients, would you please explain what spiritual needs did your patients have?
2. What were the spiritual needs of patients with different religions, cultures, and languages?
3. How did you respond to the spiritual needs of these patients?
4. What conditions did you require to respond better to these needs?
5. What circumstances prevented you from responding to the patient’s spiritual needs?
6. What were the consequences of your reaction to the patient’s spiritual needs?

### Data analysis

The current study used MAXQDA-20 to manage data, as well as Graneheim and Lundman’s conventional content analysis method to guide analysis[23]. In the present study, each interview served as a unit of analysis. The researcher divided the text into meaning units, each of which had content and context-related words, phrases, or paragraphs. Table 3 contains examples in this regard. She then condensed meaning units, while maintaining the theme. She coded them and created subcategories, and categories. The present study obtained the theme of “Nurse’s spiritual sensitivity as a higher perception and reflection toward spiritual care” (Table 4). Although the analysis process was systematic, a back and forth movement was available between the whole and the components of the text. Table 3 summarizes the analytical procedure used for each text. Table 4 summarizes all categories and subcategories. The analysis process lasted from March 2021 to January 2022.

**Table 3:** example of qualitative content analysis process

Meaning unit	Condensation	Code	Subcategories	Categories	Theme
I believe in God, who sees the deeds of His servants every moment and He has sent prophets to guide us. Therefore, I definitely adhere to a series of principles and I try my best to pay attention to the spirituality of the sick.	The nurse sees God as a witness to their actions and pays attention to the patient’s spiritual needs	Nurse’s belief in God’s existence	Spiritual characteristics	The spiritual and professional character of the nurse	Nurse’s spiritual sensitivity as a higher perception and reflection toward spiritual care
After the CPR, we try to keep the patient’s foot facing the qibla and turn the bed, and we put a headset on the patient’s ears to listen to verses of the Qur’an and Yasin Surah.	The nurses obliged themselves to do these actions for a dying patient	The nurse’s commitment to provide care for a dying patient	Professional characteristics		
Some older ICU patients, for example, would be quite disappointed if they were conscious and did not complete their prayers.	The nurse noticed that the patient was unhappy for not completing prayers.	Patient’s dissatisfaction with not completing prayers.	Mental states of patients and their families	Perception of patients and families’ spiritual needs	
There were patients who needed open-heart surgery. The process of becoming a candidate for heart surgery was stressful for the patient. Therefore, they needed to be calmed down.	The nurse perceived the patient’s need for peace of mind	Patient’s need for peace of mind	Spiritual needs of patients and their families		
I talked to patients about their diseases and asked them how they coped with the diseases. They answered that they were satisfied with God’s plan. These diseases were not comparable to the suffering of the prophets, who were the best people in the world.	The nurse noticed the patient’s reliance on God	Patient’s reliance on God	Perception of patient’s acts of worship	Nurse’s reflection of patients’ and families’ religious beliefs	
Families of critically ill patients in the operating room stood behind the operating room, prayed, read the Quran, paid nazr, or sought intercession from Hazrat Abul Fazl Al Abbas	The nurse noticed Tawassul of high-risk patients’ families to the Holy Imams	Families’ Tawassule to the Holy Imams	Perception of families’ acts of worship		

**Table 4:** Themes, categories, and subcategories extracted from qualitative content analysis

Theme	Categories	Subcategories
Nurse’s spiritual sensitivity as a higher perception and reflection toward spiritual care	The spiritual and professional character of the nurse	Spiritual characteristics
		Professional and ethical characteristics
	Perceptions of spiritual conditions and needs of patients and their families	Mental states of patients and their families
		Spiritual needs of patients and their families
	Nurse’s reflection on religious beliefs of patients and their families	Perception of patients’ acts of worship
		Perception of families’ acts of worship

### Trustworthiness

Qualitative research uses four criteria for data trustworthiness: credibility, dependability, confirmability, and transferability [24]. The present study used several methods to increase data trustworthiness. The researcher tried to spend enough time (11 months) to collect data, continuously evaluated the data, and established a good relationship with participants. In order to collect in-depth data, the researcher tried to select participants with different characteristics (maximum variation). The participants reviewed a short report of the analyzed data (member check) to see how it reflected their experiences and attitudes. Two professional co-researchers reviewed all extracted codes and categories to ensure the data credibility. To increase the dependability, peer checking was performed to see whether their viewpoints were comparable to that of the researcher. For this purpose, two researchers experienced in qualitative research confirmed the data dependability. To improve data confirmability, the study team designed a mind map during the research process. Two members of the research team (qualitative research professionals) reviewed the transcripts of multiple interviews, as well as the extracted codes and categories, to assess the data coding procedure. To improve data transferability, the researcher distributed the research findings to a number of samples who were not participants and obtained their opinions. They tried to discuss all the research stages in detail.

## Results

This qualitative content analysis aimed to explain and define the meaning, dimensions, and components of “nurse’s spiritual sensitivity” based on the participants’ experiences. The findings demonstrated a theme, three categories, and six subcategories. In addition, 497 codes remained after constant comparative analysis, condensation, and integration of codes (Table 4).

### Theme: Nurse’s spiritual sensitivity as a higher perception and reflection toward spiritual care

According to the participants’ experiences, “nurse’s spiritual sensitivity as a higher perception and reflection toward spiritual care” included three categories of “the spiritual and professional character of the nurse”, “nurse’s perception of the patient’s spiritual conditions and needs” and “nurse’s reflection on religious beliefs of patients and their families”.

### The spiritual and professional character of the nurse

Most of the participants in this study reported that a spiritually sensitive nurse had some personality traits, including spiritual, moral, and professional characteristics.

#### Spiritual characteristics

According to the participants’ experiences, the spiritual characteristics of a nurse included nurse’s belief in God existence, in God’s plan for the patient, in patient’s demands fulfilled by God, in patient’s forgiveness of sins due to illness, as well as nurse’s commitment to pray, complete payers, fast, and read the Quran for patients.

The participants acknowledged that when they recognized God in all professional situations, they did their best to discover the patient’s spiritual needs and provide a holistic care to them. They wanted to help the people in need because of all the good things God had done for them.

“I know that God is present everywhere and observes my deeds. I always remind myself that because God helped me to my position, I am obligated to assist people in need.” (Participant No. 3, a 30-year-old man with 7 years of work experience).

According to nurses, everything is based on God’s plan, and man is unaware of what God has planned for them. When something is accomplished, they realize that it has been God’s plan. Spiritually sensitive nurses see themselves as a means to fulfill God’s purpose, and help critically ill patients to understand this issue.

“A patient who had recently been diagnosed with AML was really distressed. I constantly reassured her that this illness was a test from God. I think God is concerned about his servants. Eventually, He will accomplish His plan.” (Participant No. 7, a 35-year-old Shiite woman working in the hematology department).

#### Professional and ethical characteristics

According to participants’ experiences, this subcategory included nurse’s sympathy for patients and their families, patient-nurse close relationship, nurse’s patience in providing care, nurse’s empathy with patient, nurse’s respect for patient’s beliefs, patient’s spiritual needs being satisfied by nurses regardless of their beliefs, the nurse’s attention to human dignity, the nurse’s respect for the spiritual beliefs of families, the nurse’s commitment to provide care to an end-of-life patient, nurse’s information about patient’s acts of worship, nurse’s responsibility to meet the patient’s spiritual needs.

According to the participants, nurses who feel sympathetic for patients and their families pay more attention to spiritual issues. A sympathetic nurse recognizes the pain and suffering of patients and their families and strives to relieve it.

“I believe the patient’s companion is under a lot of pressure. They must experience the emotional poverty of the sick person and manage both the economic and emotional crises. Frequently, their demands are disregarded, and they must remain accountable so long as their patient is enduring a terrible phase of sickness. No one can satisfy their own desires.” (Participant No. 2, a 41-year-old woman with 15 years of work experience).

Nurses deal with patients of different religions in this province. According to the nurses’ experiences, when a nurse is spiritually sensitive, they meet the spiritual needs of patients regardless of their beliefs. The Shiite and Sunni nurses present in this study paid equal attention to the spiritual needs of Shiite and Sunni patients.

“To me, the essence of humanity is more important than religion. Both Shiite and Sunni patients receive the same level of care.” (Participant No. 10, a 32-year-old Sunni man).

### Perception of the conditions and needs of patients and their families

Participants in this study emphasized that a spiritually sensitive nurse always received feedbacks from patients and their families. These feedbacks helped the nurse to understand the condition of patients and their families and to discover their spiritual needs. This category includes two subcategories: “mental states of patients and their families” and “spiritual needs of patients and their families”.

#### The mental states of patients and their families

Nurses participating in this study witnessed “the patient’s dissatisfaction with not doing acts of worship, the patient’s sense of loneliness, the patient’s fear of death, the patient’s grief, the patient’s regret and sense of guilt in personal life, the patient’s frustration of the course of treatment, frustration of the patient’s companion.

According to the participants in this study, a spiritually sensitive nurse notices this sense of dissatisfaction and knows that acts of worship are very important for some patients, especially the older people, and if the patient is unable to perform these acts, they will feel guilty, thus nurses pave the way for patients to perform their religious acts.

“Some older ICU patients, for example, would be quite disappointed if they were conscious and did not complete their prayers.” (Participant No. 19, a 38-year-old Sunni female nurse).

The nurses’ experiences showed that families of critically ill patients were more likely to experience despair, hopelessness, and helplessness. Therefore, a spiritually sensitive nurse paid more attention to such families.

“There was a young patient who had needed a transplant; it was a dangerous procedure, and the doctor had warned the companions that it was highly improbable that the patient would survive. His family was utterly disappointed.” (Participant No.18, a 34-year-old Shiite woman).

#### The spiritual needs of patients and their families

Patients, according to nurses, needed spiritual support, religious instruction, peace of mind and they required to practice acts of worship, and patients’ families needed psychological support and peace of mind.

According to participants, a spiritually sensitive nurse realizes the patient’s need for religious instruction because they respond to disease with all their existential dimensions. Since a person’s health depends on the equilibrium between all health dimensions, it is impossible to know one’s body, mind, and social personality without spirituality.

“Some patients have to stay at hospital longer and want to complete their prayers during the time of admission. If their physical condition does not allow them to perform Wudu, we will teach them how to do it.” (Participant No. 1, a 25-year-old Shiite woman).

The participants’ experience showed that a spiritually sensitive nurse had to focus on both the patients’ and their families’ peace of mind, and tried their best to calm them. Nurses believed that families’ peace of mind was as important as that of critically ill patients.

“Families require peace of mind when hospital stays are lengthy or when a patient’s condition is so dangerous that they may die today or tomorrow during separate shifts. Therefore, we permit families to act on behalf of their patients.” (Participant No. 8, a 34-year-old woman with 11 years of work experience).

### The nurse’s reflection on the religious beliefs of patients and their families

This category includes two subcategories of “perception of patients’ acts of worship” and “perception of families’ acts of worship”.

#### Perception of patients’ acts of worship

This subcategory included “patients’ trust in God during illness, their adherence to religious ceremonies, their Tawassul to the Holy Imams, their belief in others’ demands fulfilled by God, their adherence to complete prayers, read the Quran, fast, and praise God.”

Nurses participated in this study reported that when they witnessed patients’ trust in God, they realized the patient’s beliefs and paid attention to their spirituality.

“Patients believe in God and rely on him. When I speak with them, I will see their trust. I realize their reliance on God.” (Participant No. 9, a 44-year-old woman with 20 years of work experience).

Nurses participated in this study reported patients’ Tawassul to the Holy Imams while caring for them. After observing the patients’ Tawassul to the Holy Imams, a spiritually sensitive nurse focused more on providing spiritual care.

“I saw a patient seeking intercession from the Holy Imams. I admired them for taking the Imams and Prophet as models and attempting to calm themselves via their beliefs.” (Participant No. 7, a 35-year-old Shiite woman).

#### Perception of families’ acts of worship

Families’ acts of worship included their Tawassul to the Holy Imams, their reliance on God, their adherence to read the Quran on the patient’s bedside, their adherence to do acts of worship for a dying patient, their beliefs in others’ demands fulfilled by God.

According to participants, families seek intercession from the Holy Imams for recovery of their critically ill patients. A spiritually sensitive nurse sees families’ Tawassul and tries to meet their spiritual needs.

“Families of critically ill patients stood behind the operating room, prayed, read the Quran, paid nazr, or sought intercession from Hazrat Abul Fazl Al Abbas.” (Participant No. 9, a 44-year-old woman with 20 years of work experience).

The participants in the study acknowledged that families relied on God when their patients’ recovery was beyond the power of the healthcare system. When a nurse notices families’ reliance on God and their praying, she becomes sensitive to the families’ spiritual needs.

“One of these patients was someone I know. I spoke with his family members, all of whom were distressed since their loved one’s situation was not at all favorable. The physician had given up hope that the patient would recover. Even though the patient’s mother was discouraged, she relied on God and continued to pray for her kid.” (Participant No. 9, a 44-year-old woman with work experience in general wards).

## Discussion

This study aimed to explain Iranian nurses’ experiences of spiritual sensitivity. Based on the participants’ experiences, spiritually sensitive nurses could meet patients and families’ spiritual and religious needs via their attention to the verbal and nonverbal feedback of those they cared for. The concepts of “The spiritual and professional character of the nurse,” “nurse’s perception of the patient’s spiritual conditions and needs” and “nurse’s reflection on religious beliefs of patients and their families” were examples of nurses’ spiritual sensitivity in the workplace.

The spiritual and personal character of the nurse confirms their spiritual and professional characteristics. Based on the participants’ experiences, spiritual characteristics included nurse’s commitment to religious duties and belief in God’s existence and plan for the patient’s destiny. Batstone et al. (2020) also acknowledged that nurses’ beliefs helped them to meet their patients’ spiritual needs and give them spiritual care [25]. Other studies suggested that spiritually sensitive nurses were more likely to provide spiritual care [26, 27]. Another study showed that when a nurse prayed for a patient, they trusted in God’s plan for the patient’s future [28]. The participants’ experiences also showed that a nurse’s ability to develop spiritual sensitivity could be attributed to a variety of professional characteristics, such as sympathy for patients and their families, empathy, patience, one’s own role in meeting patients’ spiritual needs, and an appreciation for their inherent dignity. An Iranian study showed that nurse’s moral characteristics, friendliness and communication skill, accountability and vitality played a role in the nurse’s perception of the patient’s spiritual needs and provision of spiritual care [27]. Other studies indicated that the nurse’s respect for the patient’s spiritual beliefs and dignity were effective in providing spiritual care for the patient [29, 30]. A nurse’s spiritual care for patients and their families does not always imply that the nurse is spiritually sensitive. External factors, such as organizational regulations, superior satisfaction, the need to make patients satisfied, and the desire to promote organizational position affect spiritual care provision. A person’s spiritual sensitivity stems from inside and paves the way for voluntary provision of spiritual care. The results of this study showed that religion was ineffective in spiritual sensitivity, but the results of the other studies showed that religion did affect spiritual care.

The second sign of spiritual sensitivity in a nurse is to perceive the spiritual conditions and needs of patients and their families. According to the participants’ experiences, the mental states of patients and their families, such as feelings of loneliness, grief, fear of death, and dissatisfaction with not conducting acts of worship influenced the spiritual sensitivity of nurses. A study showed that nurses’ attention to the mental states of patients and their families led them to find and address patients’ spiritual needs [31]. In addition, according to the participants’ experiences, spiritual needs of patients and their families, including the need for spiritual support and peace of mind, affected nurses’ spiritual sensitivity. Nurses must first identify these needs to provide spiritual care. A study showed that families of dying patients required spiritual support and peace of mind, which were ignored by some nurses [32]. Although nurses in the present study emphasized the importance of the spiritual needs of patients and their family members, studies revealed that healthcare professionals and nurses still paid little attention to patients’ religious and spiritual needs compared to their needs for acute medicine [33, 34]. All nurses were unable to consider the mental states and spiritual needs of patients and their families in different situations. However, nurse’s spiritual sensitivity addressed these mental states and spiritual needs and provided holistic care, which was evaluated in the present study as a dimension of spiritual sensitivity in nurses. Other studies mentioned this concept using terms such as intuitive sense [31], while the present study examined this concept deeply, leading to an in-depth perception of this concept.

The third concept was the nurse’s reflection on the religious beliefs of patients and their families, such as trusting in God and adhering to religious duties. Perceiving patients’ spiritual differences enables nurses to respond to their spiritual needs [35]. Cruz et al. (2018) found that nurses’ perception of the patient’s spiritual needs allowed them to provide care for patients according to their spiritual values ​​and beliefs [36]. According to the findings of the present study, a spiritually sensitive nurse understands and meets families’ spiritual needs. This finding is consistent with that of other studies [37, 38]. Therefore, nurses should try to meet patients’ spiritual needs based on that of patients [39]. Patients’ religious differences and spirituality are effective in spiritual care provided by a nurse. The stronger the religious beliefs of patients and their families, the more attention the nurse pays to the patients’ spiritual needs. The present study showed that spiritual sensitivity was provided voluntarily and regardless of the patients’ religious beliefs.

According to Iranian legislation, being a Muslim is one of the requirements for employment in many positions, hence all participants in the current study were Muslims. We were unable to obtain data from non-Muslim nurses, and we were limited in what we could do. Due to the religious and cultural background of Iran and the fact that the phenomenon being studied is closely tied to religion, more quantitative and qualitative research in other social and cultural contexts is needed to confirm the findings of this study.

## Conclusion

The study results showed that the nurse’s spiritual sensitivity is internal, causing the nurse to pay more attention and seek the spiritual needs of patients and their families. As a result, spiritual sensitivity leads nurses to provide holistic care. Nurses’ spiritual sensitivity is difficult to assess and generally, the provision of spiritual care is rarely a reason for spiritual sensitivity in the nurse and spiritual sensitivity comes before the provision of spiritual care. Therefore, spiritual sensitivity should be institutionalized in nursing students and curricula should focus on the spiritual sensitivity of nurses. Nurse managers should evaluate the spiritual sensitivity of nurses to find and eliminate the shortcomings of holistic care provision. In addition, to confirm the study findings, more quantitative and qualitative research should confirm the results in other social and cultural contexts, especially in non-religious ones.

## Data Availability

The datasets used for the current study are available from the corresponding author upon request.
